# A young patient with systemic lupus erythematosus and chest pain

**DOI:** 10.1093/ehjcr/ytae081

**Published:** 2024-02-10

**Authors:** Andreas Y Andreou, Jovana Strika, Theodoros Ntoskas, Demetra Nikiforou

**Affiliations:** Department of Cardiology, Limassol General Hospital, Nikeas street, Pano Polemidia, PO Box 56060, 3304 Limassol, Cyprus; Department of Basic and Clinical Sciences, University of Nicosia Medical School, 93 Agiou Nikolaou Street, Engomi, 2408 Nicosia, Cyprus; Department of Cardiology, Limassol General Hospital, Nikeas street, Pano Polemidia, PO Box 56060, 3304 Limassol, Cyprus; Department of Cardiology, Mediterranean Hospital of Cyprus, Limassol, Cyprus; Department of Rheumatology, Limassol General Hospital, Limassol, Cyprus

A 21-year-old man, a current some day smoker with a history of arterial hypertension, mixed hyperlipidaemia, and systemic lupus erythematosus (SLE) since the age 14, presented with 24 h of chest discomfort at rest. Systemic lupus erythematosus has been diagnosed in the presence of malar rash, diffuse (class IV) lupus nephritis, lymphopaenia, and abnormal titres of anti–double-stranded DNA antibodies (998 IU/mL; normal < 30 IU/mL). The patient was receiving treatment for SLE with mycophenolate mofetil (1.0 g bid) combined with hydroxychloroquine (200 mg b.i.d.) and prednisolone (5 mg o.d.) prior to his recent admission. However, he was not compliant with drug therapy and had moderately active SLE [SLEDAI-2000 score: 8; pyouria (50/μL) unrelated to urinary tract infection or asymptomatic bacteriuria and persistent proteinuria (1785 mg/24 h)]. Furthermore, he denied having any viral respiratory or gastrointestinal infection prodrome, and on admission, he was afebrile with nothing remarkable on physical examination. The admission electrocardiogram (ECG) is shown in *Figure 1A*. Bedside echocardiography showed normal left ventricular ejection fraction, hypokinaesia of the apical septum and apical inferior wall, and mild pericardial effusion. Serum troponin I level on admission was 0.58 ng/mL (normal 0.00–0.20 ng/mL). The non-concave ST-segment elevation morphology observed in leads II, aVF, and III in association with reciprocal ST-segment depression in lead aVL favoured acute myocardial infarction (MI).^1,2^ Emergency coronary angiography revealed a proximal ‘wrap-around’ left anterior descending (LAD) artery culprit lesion (*Figure 1B*; see Supplementary material online, *Video S1*) successfully tackled by stent angioplasty (see Supplementary material online, *FigureS1*). Serum troponin I levels increased to 10.68 ng/mL after angioplasty. Furthermore, serum C-reactive protein levels were measured at 8.10 mg/L (normal 0.00–5.00 mg/L) on the second day after admission. The activated partial thromboplastin clotting time-based lupus ratio test was positive (2.05; reference 1.00–1.40), but on repeat examination 3 months later, the test was negative, thereby precluding antiphospholipid syndrome. The patient underwent cardiac magnetic resonance imaging (CMR) 4 days after admission, which showed bright signal intensity in the apical septum on T_2_- short-tau inversion recovery (STIR) images reflecting myocardial oedema and transmural late gadolinium enhancement (LGE) in the apical septum and apical inferior wall (*Figure 1C–F*), thereby confirming MI, most likely due to microembolization from the culprit lesion.^3^ Furthermore, the bright signal intensity noted in the mid inferolateral wall on T_2_-STIR images disappeared on repeat CMR performed 8 weeks after admission, without evidence of LGE (*Figure 1G* and *H*), thereby suggesting myocarditis-related oedema most likely owing to SLE, as opposed to SLE-related vasculitis affecting the small coronary arteries, which would have resulted in subendocardial LGE.^4^ A follow-up ECG (see Supplementary material online, *FigureS2*) showed complete resolution of ST-segment elevation with evolution of negative T-waves. The patient was discharged home after a 5-day uneventful hospital stay and remained asymptomatic at 1-year follow-up. He was treated with dual antiplatelet therapy consisting of aspirin and ticagrelor for 1 year, followed by monotherapy with aspirin. The patient was instructed to adhere to the same medication regimen as the one prescribed before his recent admission due to the absence of clinical and serological evidence of SLE activity. About 1 year after his recent admission, the patient underwent renal biopsy due to worsening proteinuria (4474 mg/24 h) revealing focal (class III) lupus nephritis (activity index score 6 and chronicity index score 1). Therefore, he received induction (1.5 g b.i.d.) and then maintenance treatment with mycophenolate mofetil combined with hydroxychloroquine and prednisone.

**Figure 1:**
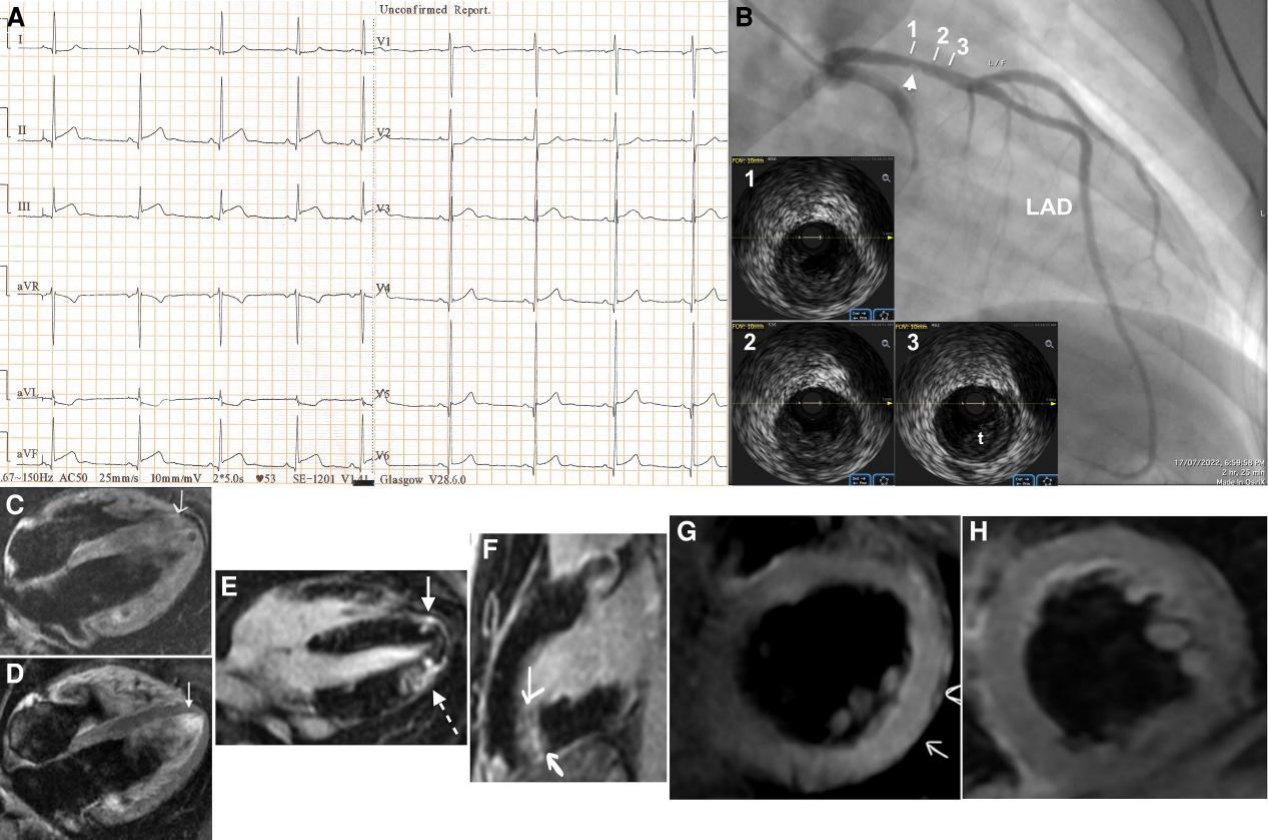
(*A*) Twelve-lead electrocardiogram on admission showing >0.1 mV obliquely straight ST-segment elevation at the J point in leads II, aVF, and III, concave ST-segment elevation in leads V3–V6 associated with an end-QRS notch in leads V4–V6, and ST-segment depression in leads aVL, I, and V2. (*B*) Conventional coronary artery angiographic image depicting a hazy, obstructive culprit lesion (arrowhead) in the proximal segment of a wrap-around left anterior descending artery. Cross-sectional intravascular ultrasound images at the site of culprit lesion (embedded panels 1, 2, and 3) showing a complicated eccentric, hypoechoic (soft) plaque with probable disruption (embedded panel 1) and subacute thrombus (embedded panel 3: t) recognized by its light to dark grey appearance with white speckles. (*C–H*) Cardiac magnetic resonance imaging. Bright signal intensity in the apical septum (*C*; arrow) owing to myocardial oedema, which disappeared (*D*; arrow) on repeat cardiac magnetic resonance imaging 8 weeks after admission. Transmural late gadolinium enhancement in the apical septum and apical inferior wall (*E* and *F*; arrows) owing to myocardial infarction. Sub-epicardial late gadolinium enhancement in the apical lateral wall (*E*; dotted arrow) in the absence of myocardial oedema (*C*) ascribed to old myocarditis. Bright signal intensity in the mid inferolateral wall (*G*; arrows), which disappeared on repeat cardiac magnetic resonance imaging (*H*) without evidence of late gadolinium enhancement; it was ascribed to systemic lupus erythematosus myocarditis-related oedema.

This case illustrates a challenging concomitant diagnosis of acute atherosclerosis-related MI and acute myocarditis in a patient with SLE and highlights the role of ECG interpretation in choosing invasive coronary angiography for initial patient investigation and the complementary role of CMR in making the diagnosis.

## Supplementary Material

ytae081_Supplementary_Data

## Data Availability

The data underlying this article are available in the article and in its online supplementary material.

## Supplementary material

**Consent:** The patient has provided written and verbal consent for his images and case details to be published in compliance with COPE guidelines.

**Funding:** None declared.

## References

[1] Brady WJ , Syverud SA, Beagle C, Perron AD, Ullman EA, Holstege C, et al Electrocardiographic ST-segment elevation: the diagnosis of acute myocardial infarction by morphologic analysis of the ST segment. Acad Emerg Med 2001;8:961–967. 11581081 10.1111/j.1553-2712.2001.tb01094.x

[2] Bischof JE , Worrall C, Thompson P, Marti D, Smith SW. ST depression in lead aVL differentiates inferior ST-elevation myocardial infarction from pericarditis. Am J Emerg Med 2016;34:149–154. 26542793 10.1016/j.ajem.2015.09.035

[3] Kim BG , Kim KH, Nah JC, Cho SW. Simultaneous left and right ventricular apical thrombi after occlusion of the wrapped left anterior descending artery. J Cardiol Cases 2019;19:153–156. 31073347 10.1016/j.jccase.2018.12.015 PMC6495048

[4] Mavrogeni S , Bratis K, Markussis V, Spargias C, Papadopoulou E, Papamentzelopoulos S, et al The diagnostic role of cardiac magnetic resonance imaging in detecting myocardial inflammation in systemic lupus erythematosus. Differentiation from viral myocarditis. Lupus 2013;22:34–43. 23035042 10.1177/0961203312462265

