# Supplementary figures and images for: A young patient with systemic lupus erythematosus and chest pain
Source: Eur Heart J Case Rep. 2024 Feb 10;8(2):ytae081. doi: 10.1093/ehjcr/ytae081 (PMC10894011; doi:10.1093/ehjcr/ytae081)

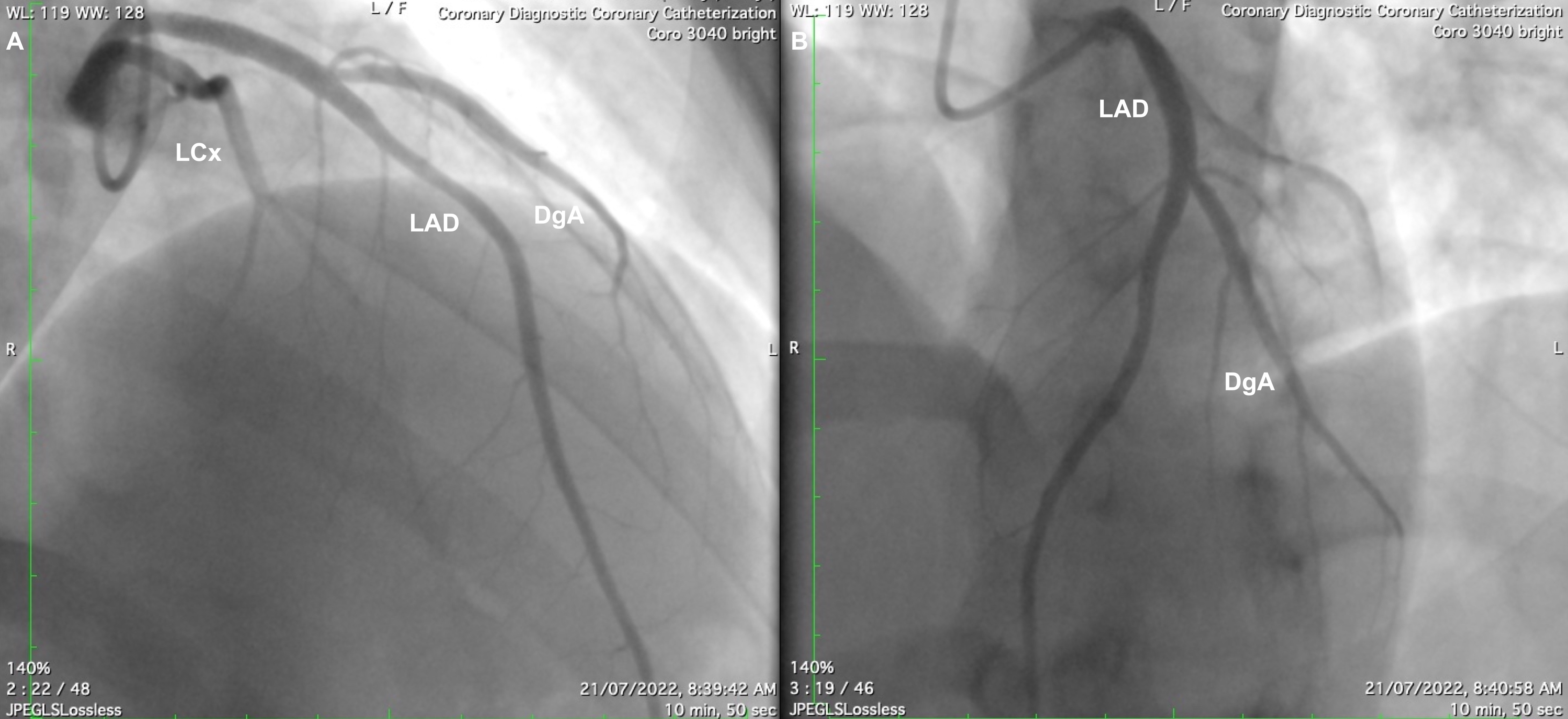

Supplement: ytae081_Supplementary_Data [file ytae081_supplementary_data.zip › Figure 1.tiff]

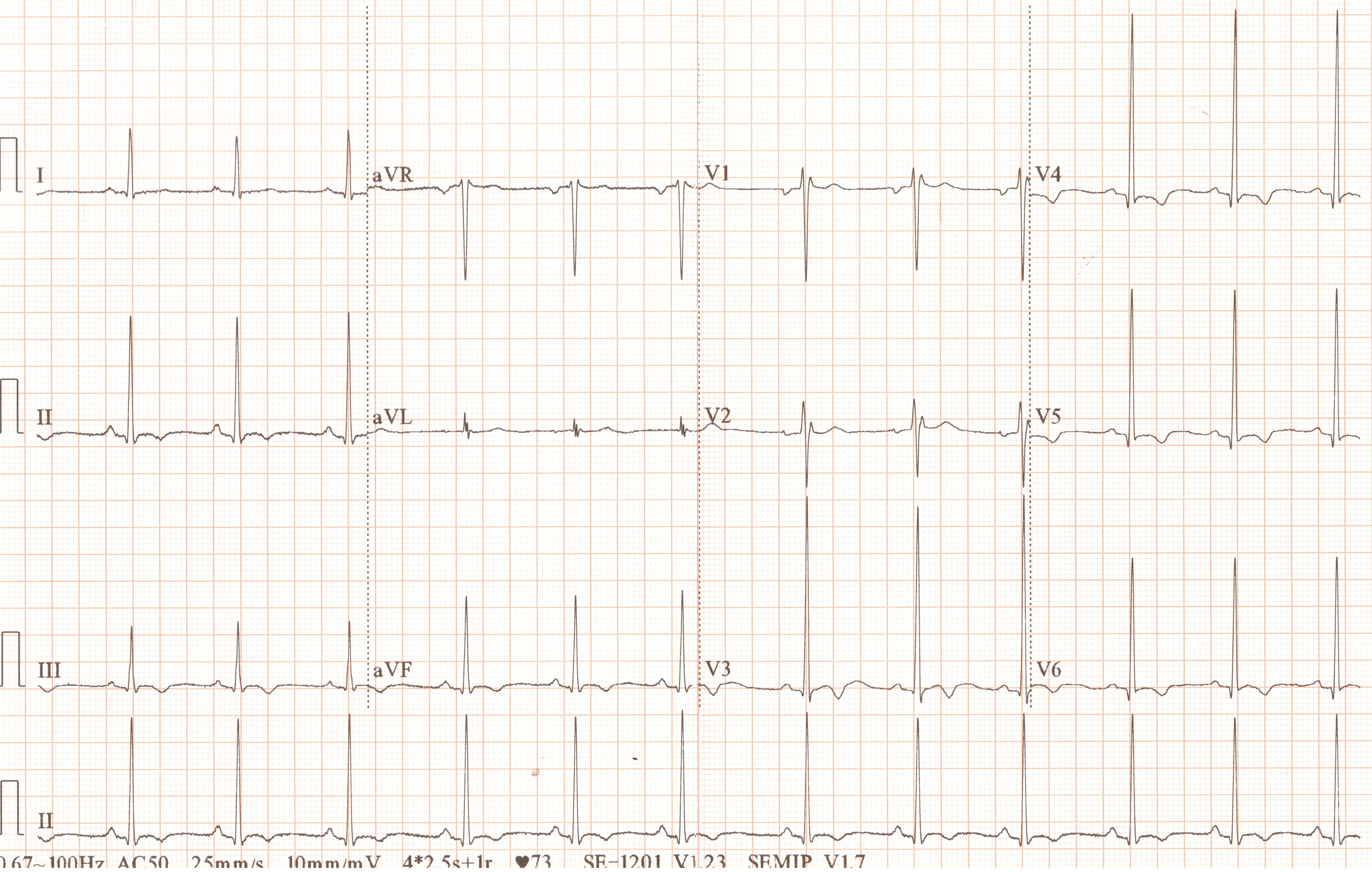

Supplement: ytae081_Supplementary_Data [file ytae081_supplementary_data.zip › Figure 2.tiff]
